# (Meta)Genomic Analysis Reveals Diverse Energy Conservation Strategies Employed by Globally Distributed *Gemmatimonadota*

**DOI:** 10.1128/msystems.00228-22

**Published:** 2022-08-01

**Authors:** Xiaowei Zheng, Xin Dai, Yaxin Zhu, Jian Yang, Hongchen Jiang, Hailiang Dong, Li Huang

**Affiliations:** a State Key Laboratory of Microbial Resources, Institute of Microbiology, Chinese Academy of Sciences, Beijing, P.R. China; b College of Life Sciences, University of Chinese Academy of Sciences, Beijing, P. R. China; c State Key Laboratory of Biogeology and Environmental Geology, China University of Geosciences, Wuhan, P.R. China; d State Key Laboratory of Biogeology and Environmental Geology, China University of Geosciences, Beijing, P. R. China; University of British Columbia

**Keywords:** metagenome, *Gemmatimonadota*, anoxygenic phototrophs, photosynthesis gene cluster, physiology, phylogeny

## Abstract

*Gemmatimonadota* is a phylum-level lineage distributed widely but rarely reported. Only six representatives of *Gemmatimonadota* have so far been isolated and cultured in laboratory. The physiology, ecology, and evolutionary history of this phylum remain unknown. The 16S rRNA gene survey of our salt lake and deep-sea sediments, and Earth Microbiome Project (EMP) samples, reveals that *Gemmatimonadota* exist in diverse environments globally. In this study, we retrieved 17 metagenome-assembled genomes (MAGs) from salt lake sediments (12 MAGs) and deep-sea sediments (5 MAGs). Analysis of these MAGs and the nonredundant MAGs or genomes from public databases reveals *Gemmatimonadota* can degrade various complex organic substrates, and mainly employ heterotrophic pathways (e.g., glycolysis and tricarboxylic acid [TCA] cycle) for growth via aerobic respiration. And the processes of sufficient energy being stored in glucose through gluconeogenesis, followed by the synthesis of more complex compounds, are prevalent in *Gemmatimonadota*. A highly expandable pangenome for *Gemmatimonadota* has been observed, which presumably results from their adaptation to thriving in diverse environments. The enrichment of the Na^+^/H^+^ antiporter in the SG8-23 order represents their adaptation to salty habitats. Notably, we identified a novel lineage of the SG8-23 order, which is potentially anoxygenic phototrophic. This lineage is not closely related to the phototrophs in the order of *Gemmatimonadales*. The two orders differ distinctly in the gene organization and phylogenetic relationship of their photosynthesis gene clusters, indicating photosystems in *Gemmatimonadota* have evolved in two independent routes.

**IMPORTANCE** The phylum *Gemmatimonadota* is widely distributed in various environments. However, their physiology, ecology and evolutionary history remain unknown, primary due to the limited cultured isolates and available genomes. We were intrigued to find out how widespread this phylum is, and how it can thrive under diverse conditions. Our results here expand the knowledge of the genetic and metabolic diversity of *Gemmatimonadota*, and shed light on the diverse energy conservation strategies (i.e., oxidative phosphorylation, substrate phosphorylation, and photosynthetic phosphorylation) responsible for their global distribution. Moreover, gene organization and phylogenetic analysis of photosynthesis gene clusters in *Gemmatimonadota* provide a valuable insight into the evolutionary history of photosynthesis.

## INTRODUCTION

Bacteria of the phylum *Gemmatimonadota* (formerly *Gemmatimonadetes* [1], KS-B division [2], or BD group [3]) are abundant (0.2 to 6.5% of the total bacterial population by 16S rRNA gene counts) in various habitats, such as waste water, agricultural soil, fresh water, and forest soil (4–9). The *Gemmatimonadota* phylum comprises five groups at the class level (groups 1–5) according to 16S rRNA gene sequence surveys (10, 11). Only six strains belonging to this phylum have been cultivated and characterized to date, and they are Gemmatimonas aurantiaca T-27 (1), *Gemmatirosa kalamazoonesis* KBS708 (12), Gemmatimonas phototrophica AP64 (13), Longimicrobium terrae CB-286315 (14), *Roseisolibacter agri* AW1220 (11), and *Gemmatimonas groenlandica* TET16 (15). All of these strains except *L. terrae* CB-286315 belong to the class *Gemmatimonadetes* (group 1). *L. terrae* CB-286315 is affiliated with the class *Longimicrobia* (group 3). No isolates or metagenome-assembled genomes (MAGs) belonging to the other three classes (groups 2, 4, and 5) have been reported. Although *Gemmatimonadota* are generally believed to play significant roles in biogeochemical cycles (13, 16–20), very little is known about this group of organisms.

In this study, we obtained 12 and 5 high-quality *Gemmatimonadota* MAGs from the Qinghai Lake sediments and the South Indian Ocean sediments, respectively, and examined the genetic and metabolic diversity of these MAGs, along with the genomes and MAGs of *Gemmatimonadota* from the public databases. *Gemmatimonadota* have evolved diverse modes of energy conservation, including aerobic respiration, fermentation, and anoxygenic photosynthesis, in agreement with their widespread presence. We identified a novel phototrophic lineage (SG8-23 order), which is closely related to neither *G. groenlandica* TET16 nor *G. phototrophica* AP64, the only two known phototrophic *Gemmatimonadota* isolates in the *Gemmatimonadales* order (13, 15, 21, 22). The two orders differ distinctly in the gene organization of their photosynthesis gene clusters (PGCs). Phylogenetic analysis based on bacteriochlorophyll biosynthesis genes (*acs*F and *bch*H) and photosynthetic reaction center subunits (*puf*L and *puf*M), shed significant light on the two independent evolutionary routes of photosynthesis in *Gemmatimonadota*.

## RESULTS AND DISCUSSION

### Biogeography of *Gemmatimonadota*.

**FIG 1:**
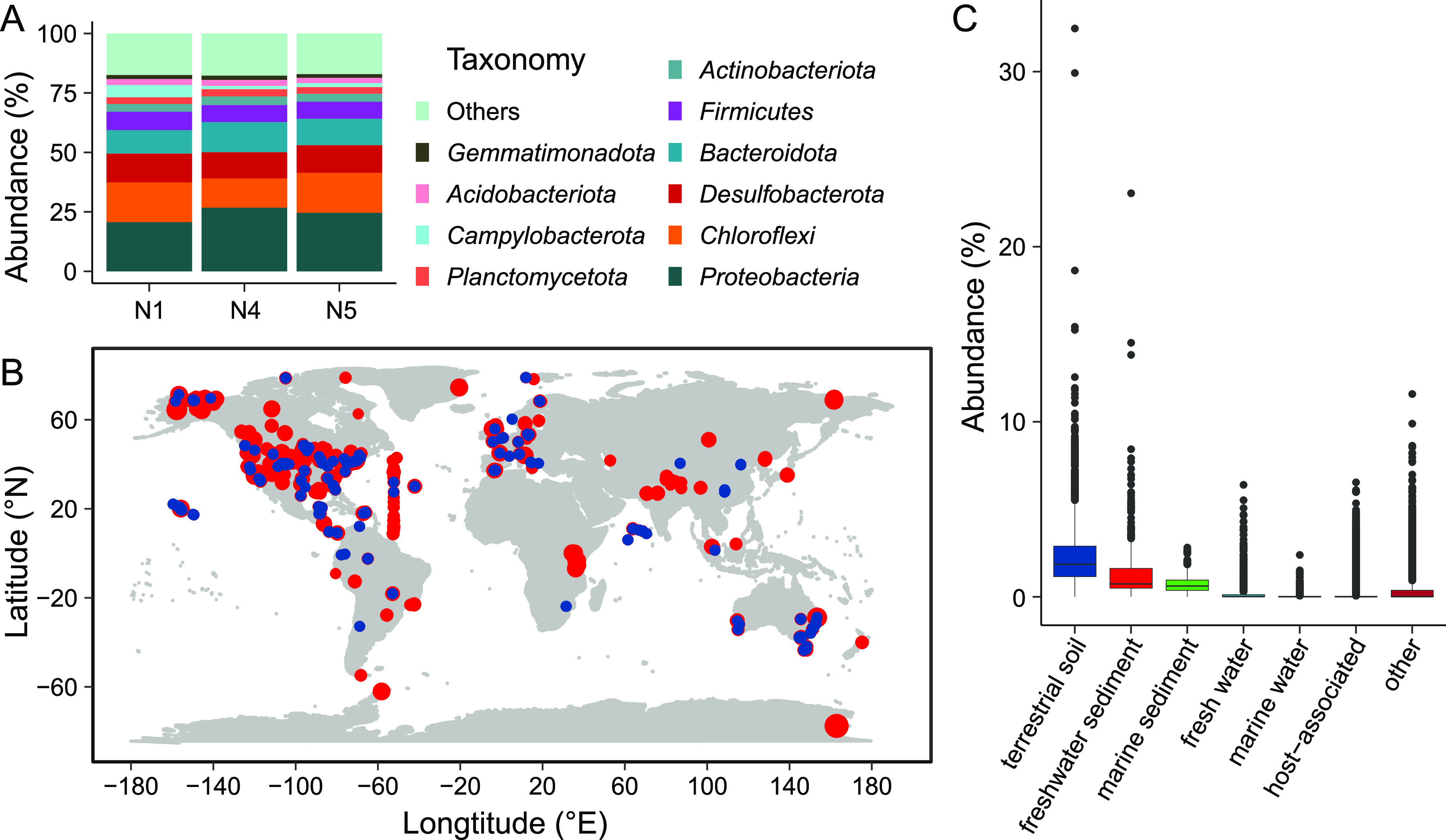
Distribution of *Gemmatimonadota*. Taxonomic composition of organisms from the sediment samples based on 16S _mi_tags at the phylum level (A). Distribution of *Gemmatimonadota* around the world (B). Relative abundance (%) of *Gemmatimonadota* in different habits based on 16S rRNA genes (C). Red and blue circles represent samples from those locations that contain *Gemmatimonadota* or not, respectively.

A total of 0.7 billion paired-end (PE) raw sequencing reads (~170 Gb) from the three Qinghai Lake sediment samples were obtained (Data Set S1, Sheet 1 in the supplemental material). Initial assembly with SPAdes produced contigs (≥1,000 bp) of 1.86, 1.89, and 1.92 Gb in total length for N1, N4, and N5, respectively (Data Set S1, Sheet 1). A total of 122,010 16S rRNA gene tags were extracted from these metagenomes (16S _mi_tags), and 15,924 operational taxonomic units (OTUs) were obtained by clustering the 16S _mi_tags against the SILVA database. By taxonomic assignment based on the SILVA taxonomy, the obtained OTUs belong to at least 33 phyla. Bacteria dominated the microbial community with a relative abundance of 92.15–93.58%, with *Proteobacteria* (20.71–26.78%) and *Chloroflexi* (12.31–16.78%) being the top two most abundant phyla (Fig. 1A and Data Set S2, Sheet 1). Notably, the relative abundance of phylum *Gemmatimonadota* was about 1.55–1.79%, and is similar to that in the deep-sea sediments (1.15–4.39%) of the Southwest Indian Ocean (23).

To learn more about the global distribution of *Gemmatimonadota*, we retrieved 23,323 qualified samples from the Earth Microbiome Project (EMP). Over 64% (15,058) of these samples contain species belonging to *Gemmatimonadota*, and they are distributed around the globe in a variety of habitats, such as tundra, cropland, lakes, marine, and the human gut (Fig. 1B and Data Set S2, Sheet 2). The abundance of *Gemmatimonadota* varies considerably among the habitats and among samples from the same habitat. This phylum appears more abundant in tundra and permafrost, with the highest abundance of ~1/3 detected in a tundra biome, than in fresh water and marine waters (Data Set S2, Sheet 2). The median abundances of *Gemmatimonadota* are 1.85, 0.73, 0.61, 0.019, and 0.003% in samples from terrestrial soil (mostly in the permafrost zone), freshwater sediment, marine sediment, fresh water, and marine water, respectively (Fig. 1C). *Gemmatimonadota* has also been detected in trace amounts in host-associated environments such as rhizosphere, biofilm, sebum, saliva, mucus, and feces.

### Genome-based taxonomic analysis of *Gemmatimonadota*.

**FIG 2:**
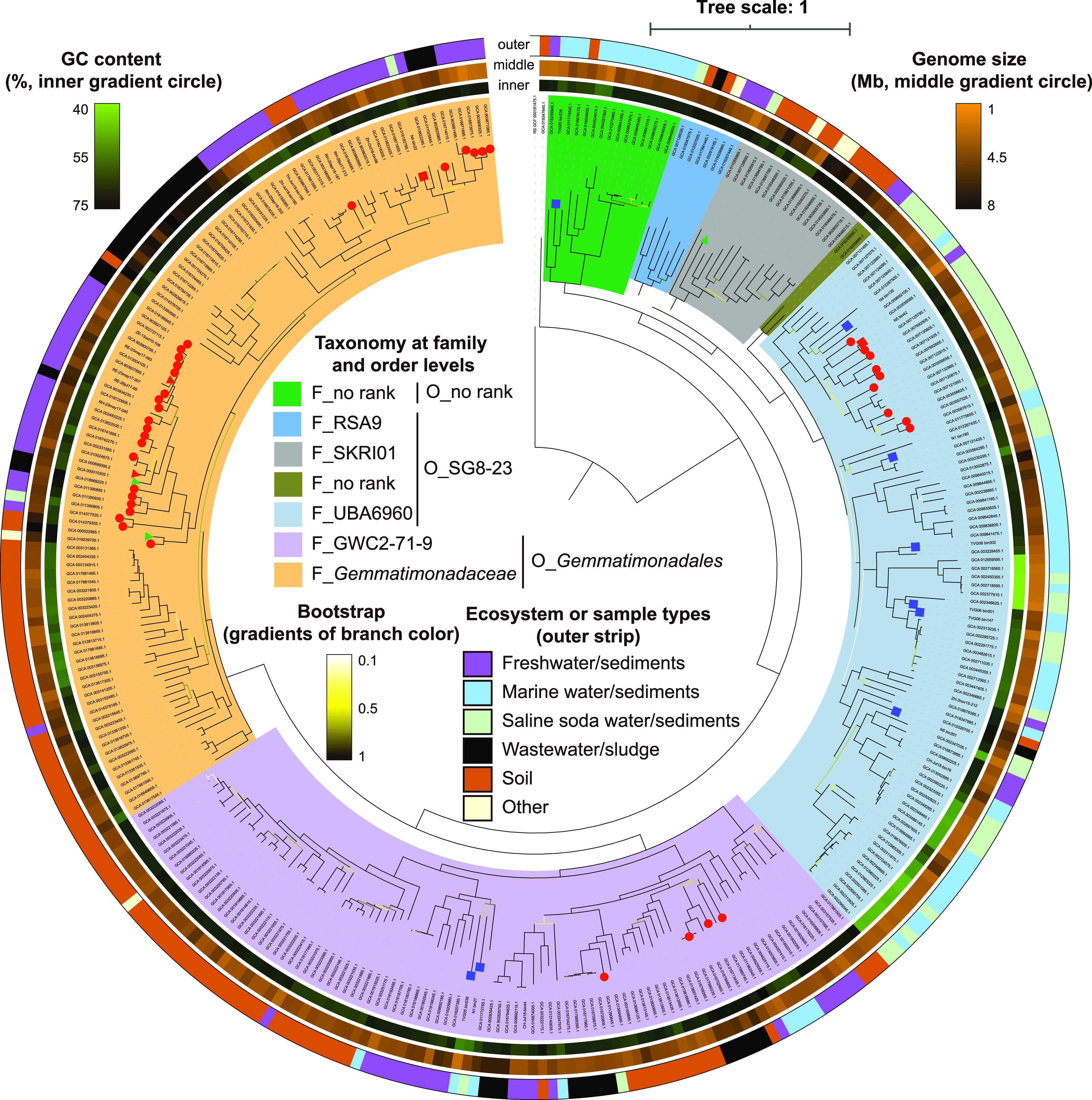
Phylogenomic tree of *Gemmatimonadota* inferred from 120 concatenated marker proteins. The tree was rooted with the genome of Gimesia maris DSM 8797 (GenBank assembly no. GCA_000181475.1). Phototrophic *Gemmatimonadota* are mark by red circles, squares, and triangles on the tip of branches. The red circles represent those genomes containing a photosynthesis gene cluster (PGC). Red and blue squares represent those MAGs we retrieved in this study containing PGC or not, respectively. Red and green triangles represent those cultured isolates containing PGC or not in their genomes, respectively. *Gemmatimonadota* clusters at family and orders are marked by different colors. The strength of support for internal nodes (bootstrap) is shown through branch colors. Inner, middle, and outer circles around the tree represent GC content (%), genome size (Mb), and habitat types of these 326 *Gemmatimonadota* genomes, respectively. Details about all the 326 *Gemmatimonadota* genomes can be found in Table S1.

We assembled 12 *Gemmatimonadota* MAGs (≥75% completeness and ≤5% contamination) from the metagenomic DNA of Qinghai Lake sediments (Data Set S1, Sheet 2). Five additional MAGs were obtained from the metagenomes of deep-sea sediment samples taken from the Southwestern Indian Ocean (23). To facilitate the phylogenetic analysis of phylum *Gemmatimonadota*, we downloaded 504 medium- to high-quality *Gemmatimonadota* genomes and MAGs (≥50% completeness and ≤5% contamination) from the NCBI, GTDB, and Figshare databases (Table S1). After deduplication and quality control, 326 nonredundant genomes at strain levels (>99% ANI) were retained. These genomes ranged from 1.52 to 7.47 Mb in size with a median value of 3.57 Mb, and their GC contents were between 44.92% and 74.33% with a median of 67.37% (Fig. 2 and Table S1). The large variation in estimated genome size and GC content may imply considerable diversity within phylum *Gemmatimonadota*. Based on genome similarity and phylogenomic analysis, these nonredundant genomes are grouped into 265 potential species after dereplication using dRep v2.3.2 (24) at 95% ANI, all of which belong to class *Gemmatimonadetes* (Fig. 2 and Table S1). By GTDB classification, the 326 genomes are divided into three orders, i.e., *Gemmatimonadales* (199 genomes), SG8-23 (111 genomes), and an unclassified order (16 genomes). We focused our comparative genomic analysis on genomes from *Gemmatimonadales* and SG8-23, as the majority of *Gemmatimonadota* genomes used in this study were affiliated with these two orders. Separation of the two orders was further demonstrated by a genome-scale gene correlation analysis (Fig. S1).

### Metabolic potential of *Gemmatimonadota*.

Pangenome analysis (Fig. S2) reveals a highly expandable pangenome for *Gemmatimonadota*, which has presumably resulted from their adaptation to thriving in diverse environments. However, a hypergeometric test infers no significant differences between *Gemmatimonadales* and SG8-23 in metabolism based on KEGG functional categories (Data Set S3, Sheet 1). Therefore, we set out to construct the metabolic potentials of the entire phylum of *Gemmatimonadota* by examining functional annotations of the 326 nonredundant genomes or MAGs, to illustrate an overview of energy releasing and storing processes in this phylum.

**FIG 3:**
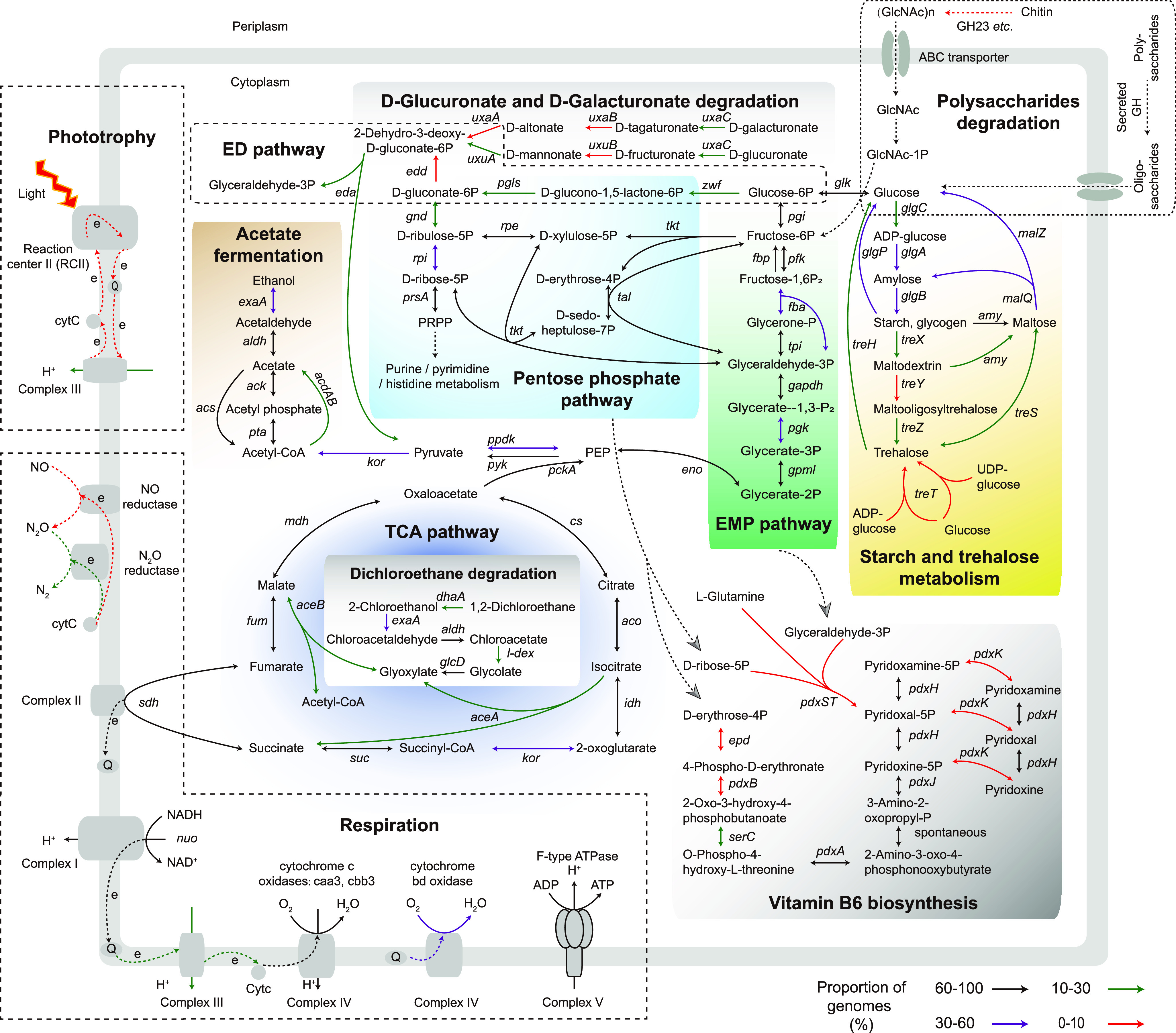
Overview of potential metabolic capabilities of *Gemmatimonadota*. Genes involved in the EMP pathway (glycolysis), gluconeogenesis, PPP (pentose phosphate pathway), ED (Entner–Doudoroff) pathway, pyruvate metabolism, TCA cycle, dichloroethane degradation, d-glucuronate and d-galacturonate degradation, vitamin B_6_ biosynthesis, starch and trehalose metabolism, respiratory chain, acetate fermentation, and membrane transporters are shown. The corresponding enzymes are represented by an ID in the figure and Data Set S4, Sheet 2. The color of the arrow represents the proportion of genomes containing the corresponding enzyme or capable of performing the metabolic reaction.

As shown in Data Set S4, Sheet 1, 129 carbohydrate-active enzyme families were observed, and members of *Gemmatimonadota* are potentially able to utilize a wide range of complex carbon sources, including chitin, starch, cellulose, and hemicellulose. The breakdown of glucose to pyruvate can be achieved through EMP (Embden–Meyerhof–Parnas, usually called glycolysis) or ED (Entner–Doudoroff) pathway in *Gemmatimonadota*, as shown in Fig. 3. Genes encoding the three key rate-limiting enzymes in glycolysis, i.e., hexokinase (*glk*), 6-phosphofructokinase (*pfk*), and pyruvate kinase (*pyk*), are annotated in 77–83% of the 326 genomes, and at least 184 genomes (56%) contain all three of these genes, showing glycolysis is a main process for breaking down glucose to pyruvate (Fig. 3 and Data Set S4, Sheet 2). Those MAGs without glycolysis may be due to the incompleteness of their genomes, and they may acquire pyruvate via the ED pathway as an alternative. For example, N1_bin27 is identified as having the key enzyme of 2-dehydro-3-deoxyphosphogluconate aldolase (*eda*) in ED pathway, indicating this MAG may obtain pyruvate without having to go through glycolysis. However, the predicted *eda* gene is found in only 50 *Gemmatimonadales* (15%) and 6 SG8-23 genomes (2%) (Data Set S4, Sheet 2), implying that the ED pathway is not commonly used for acquiring pyruvate by *Gemmatimonadota*. It was noticed that the pathway for the conversion of D-glucuronate and D-galacturonate into 2-Dehydro-3-deoxy-D-gluconate-6-phosphate exists in 10% of the 326 genomes (Data Set S4, Sheet 2). This process may play a role in replenishing the ED pathway with intermediate substrates, especially for those *Gemmatimonadota* lacking the *edd* gene, which encodes phosphogluconate dehydratase (Fig. 3). Genes encoding the three key rate limiting enzymes of the TCA cycle, i.e., citrate synthase (*cs*), aconitate hydratase (*aco*), and malate dehydrogenase (*mdh*), are annotated in most of the 326 genomes (83%, 73%, and 82% genomes, respectively), indicating that *Gemmatimonadota* acquire energy mainly through the TCA pathway and most likely live a heterotrophic lifestyle. Besides, 1,2-Dichlroroethane, a widely used chlorinated solvent with potential to harm the environment and human health, could also be degraded by *Gemmatimonadota* into glyoxylate, which can be incorporated in the TCA cycle for subsequent energy conservation (Fig. 3). This observation partially explains why some *Gemmatimonadota*, especially family *Gemmatimonadaceae*, whose genomes are enriched with benzoate and styrene degradation genes (Data Set S3, Sheet 5), are detected in contaminated environments, such as bioreactor sludge and waste water (Table S1).

The pentose phosphate pathway (PPP) provides an alternative to glycolysis for glucose oxidation (Fig. 3). Genes encoding glucose-6-phosphate dehydrogenase (G6PD, *zwf*) and 6-phosphogluconate dehydrogenase (PGD, *gnd*), two rate-limiting enzymes during the oxidative phase of PPP, are annotated in 23% and 27% of the 326 genomes, respectively. Genes for putative transketolase (*tkt*), transaldolase (*tal*), and ribulose-phosphate 3-epimerase (*rpe*), responsible for the conversion of phosphorylated carbohydrates in the nonoxidative phase, are present in over 80% of the *Gemmatimonadota* genomes. Moreover, more than half (54%) of the *Gemmatimonadota* genomes appear to encode ribose-5-phosphate isomerase (*rpi*), which catalyzes the interconversion between D-ribose-5-phosphate and D-ribulose-5-phosphate. These observations show that PPP may be a prevalent pathway in *Gemmatimonadota*, providing essential substrates (e.g., NADPH and pentose phosphate) for the synthesis of nucleotides, amino acids, cofactors, and vitamins. For example, vitamin B_6_ could be synthesized using the PPP product of the D-ribose-5-phosphate or D-erythrose-4-phosphate via two non-homologous routes (Fig. 3). The biologically active form of the vitamin B_6_ group (pyridoxal, pyridoxamine, and pyridoxine), pyridoxal 5′-phosphate (PLP), is known as an essential cofactor for cellular function in all domains of life (25). PLP-dependent enzymes have been reported as being involved in diverse cellular processes, such as the biosynthesis of amino acids, sugars, lipids, and antibiotics (25–27). However, pyridoxal 5′-phosphate synthase (*pdx*ST), which can catalyze D-ribose-5-phosphate, D-glyceraldehyde-3-phosphate, and l-glutamine into PLP, has been found in only two genomes, suggesting it is not the main route for vitamin B_6_ biosynthesis. The key enzyme of another route, pyridoxine 5-phosphate synthase (*pdx*J), which can convert 1-deoxy-D-xylulose 5-phosphate and 3-amino-2-oxopropyl phosphate to pyridoxine-5-phosphate, are identified in over 80% of 326 genomes, showing that most of *Gemmatimonadota* may be capable of synthesizing PLP on their own via this route. However, erythrose-4-phosphate dehydrogenase (*epd*) and erythronate-4-phosphate dehydrogenase (*pdx*B), the first two enzymes of this route, are seldom present in *Gemmatimonadota* genomes. Fortunately, their functions might be replaced by other nonhomologous enzymes (28).

As for carbohydrate anabolism, almost 50% of 326 genomes (162) contain all three genes likely encoding the key enzymes of gluconeogenesis, phosphoenol pyruvate carboxykinase (*pck*A), fructose-1,6-bisphosphatase (*fbp*), and glucokinase (*glk*). And the proportion of genomes containing at least one of these three rate-limiting enzymes is about 73%, 76%, 79%, respectively. Therefore, the process of gluconeogenesis storing sufficient energy in glucose, followed by the synthesis of more complex compounds (e.g., glycogen and starch), should be prevalent in *Gemmatimonadota* (Fig. 3). Trehalose, known as a protective agent that helps cells adapting to cold and high-pressure habitats, may be synthesized by *Gemmatimonadota*, as has been reported previously (21). However, we found that the biosynthesis of this compound does not seem prevalent in *Gemmatimonadota* (Fig. 3). This is because the enzyme of 1-alpha-D-glucosylmutase (*tre*Y), responsible for the conversion of maltodextrin to the precursor of trehalose (maltooligosyltrehalose) (28), is present in less than 10% of *Gemmatimonadota* genomes (Data Set S4, Sheet 2). Trehalose could also be synthesized in one step using trehalose synthase (*tre*T) from the ubiquitous metabolic intermediates of D-glucose and UDP- or ADP-glucose. Nevertheless, the *tre*T gene is detected only in 14 genomes (Data Set S4, Sheet 2).

In addition, genes for acetyl-CoA synthetase (*acs*), which synthesize acetyl-CoA for energy metabolism and for the synthesis of carbohydrates, lipids, and proteins, is identified in 77% of the genomes. Therefore, heterotrophy via acetate fermentation appears to occur in *Gemmatimonadota*. And 19 genomes are found to contain genes coding for both large and small subunits of RubisCO (*rbc*LS, ribulose-bisphosphate carboxylase), a key enzyme for CO_2_ fixation in photosynthesis, indicating photoautotrophic potential in some *Gemmatimonadota*. However, no photoautotrophs or chemoautotrophs have so far been isolated in this phylum (15).

### Characteristics of the electron transport chain in *Gemmatimonadota*.

To gain a better insight into its energy conservation, we further characterized the ETC (electron transport chain) composition of *Gemmatimonadota*.

Complex I (NADH-quinone oxidoreductase) and complex II (succinate dehydrogenase), encoded by *nuo* and *sdh*, respectively, feed electrons to membrane-bound respiratory chains. Both enzymes are found in over 80% of the *Gemmatimonadota* genomes (Fig. 3 and Data Set S4, Sheet 2). Other quinone reductases are present in over half of the *Gemmatimonadota* genomes, such as proline dehydrogenase (*putB*), glycerol-3-phosphate dehydrogenase (*glpD*), and quinoprotein glucose dehydrogenase (*gcd*), which are involved in proline, glycerophospholipid, and carbohydrate metabolism, respectively, and are also primary points for electrons entering the respiratory chain in *Gemmatimonadota*. An Mrp (multiple resistance and pH) antiporter (Na^+^/H^+^ antiporter), which contains multiple subunits (MrpBCDEFG) and is responsible for the efflux of intracellular sodium ions utilizing the proton motive force across membranes, is enriched in SG8-23, as revealed by the hypergeometric test (Data Set S3, Sheets 2, 3, and 4). Since the majority of the SG8-23 genomes are retrieved from marine and saline soda habitats (Fig. 2 and Table S1), the enrichment of the Na^+^/H^+^ antiporter represents the adaptation of this group to salty habitats. It has been reported that MrpC and MrpD are homologous to membrane-embedded subunits of NuoK and NuoMN in the respiratory chain complex I (29), implying the close kinship of Na^+^/H^+^ antiporter and complex I in proton transport and energy production.

Complex III (cytochrome c reductase), also known as the bc1 complex (or b6f in photosynthetic organisms), is a key component of both the respiratory and the photosynthetic electron transport chains. Surprisingly, the cytochrome b subunit (*pet*B) of bc1 is only found in 23% of the genomes, indicating that the bc1 complex is probably not widely used in *Gemmatimonadota*. Alternative complex III (ACIII), structurally unrelated to bc1, could serve the function of bc1 (30). This complex is found in various bacteria, including *Gemmatimonadota* (30), but is identified in fewer than 10% of the genomes in this study (Data Set S4, Sheet 2). It is possible that low similarity to known ACIII proteins in the KEGG database prevents function annotation or novel complex III subunits (or modules) exist in *Gemmatimonadota* (31). It is also worth noting that complex III is not essential in respiratory chains. For instance, cytochrome bd oxidase, which reduces of oxygen to water, obtains electrons directly from quinol without the aid of complex III but does not pump protons across the membrane (32, 33), thus reducing the efficiency of energy conversion into ATP.

Three types of complex IV, the last enzymatic complex for electron transfer in an aerobic respiratory chain, have been detected. They are caa3-type heme–copper oxygen reductase (HCO), cbb3-type HCO, and cytochrome bd oxidase, which exist in 74%, 50%, and 53% of the 326 genomes, respectively, indicating that *Gemmatimonadota* mainly use O_2_ as the terminal electron acceptor. Five genomes, including that of the cultured strain *G. aurantiaca* T-27, are found to contain all three types of complex IV. Because of the much higher affinity of cbb3-type HCOs and cytochrome bd oxidase for O_2_ than caa3-type HCOs (34–36), these strains may respond efficiently to variation in O_2_ level. On the other hand, since oxygen reductase is also found in strictly anaerobic organisms, such as Desulfovibrio desulfuricans ATCC 27774 (37), the presence of the terminal oxidases may also serve to protect oxygen-sensitive enzymes when anaerobically respiring *Gemmatimonadota* are exposed to oxic conditions. Moreover, genes encoding putative nitrous oxide reductase (NosZ) and nitric oxide reductase (NorBC) are present in at least 23% and 9% of the genomes (Data Set S4, Sheet 2), respectively, suggesting they may use N_2_O and NO as terminal electron acceptors. Nitrate and nitrite are seldom used as terminal electron acceptors in *Gemmatimonadota*, as nitrate reductase (NarGHI) and nitrite reductase (NrfAH) are only identified in 2–10% of the 326 genomes. Notably, at least 30% of *Gemmatimonadota* that may be capable of performing anaerobic respiration also possess cytochrome c oxidase or cytochrome bd oxidase (Data Set S4, Sheet 3), further demonstrating their diverse energy conservation strategies for coping with oxygen varies.

In addition, *Gemmatimonadota* possess a complete set of *men-*like genes for the synthesis of menaquinone (MK, or vitamin K2), whereas genes encoding ubiquinone (UQ, or coenzyme Q) synthesis are incomplete (Data Set S4, Sheet 2), in agreement with the finding that the major respiratory quinone of the six cultured isolates is MK-8 or MK-9 (1, 11, 12, 14, 15, 22). It is speculated that UQ, usually used by aerobic organisms, appeared about 2.5 billion years ago as a strategy of life to adapt to rising oxygen levels (38, 39). On the other hand, MK exists in microorganisms living under low O_2_ or anoxic conditions (32). There are two known pathways for MK synthesis in bacteria, i.e., the “classical” (Men) pathway and the “futalosine” (Mqn) pathway (40, 41). It appears that SG8-23 preferentially employs the classical pathway, as *men*A, *men*B, and *men*D are enriched in this order, as opposed to *Gemmatimonadales* (Data Set S3, Sheet 2), which prefers the futalosine pathway. It is unclear why one pathway is preferred over the other as both pathways utilize the same precursors and function under both aerobic and anaerobic conditions (41).

### Organization of PGCs in *Gemmatimonadota*.

Several members of *Gemmatimonadota*, including novel lineages described below, are potentially able to capture light energy through photosynthetic phosphorylation for their growth (Fig. 3). Of the 326 nonredundant genomes, 43 are found to contain a ~40-kb-long photosynthesis gene cluster (PGC) (Table S1). These include genomes of *Gemmatimonadota* AP64 and TET16, the only two cultured phototrophic strains of this group (13, 15). Of the remaining 41 genomes, 22 are assembled from metagenomes obtained from fresh water, 13 from soda lakes, and 6 from other habitats. No phototrophic *Gemmatimonadota* have been detected in marine habitats. Taxonomically, 33 of the 43 genomes belong to the order of *Gemmatimonadales*, and the other 10 genomes to the UBA6960 family of the SG8-23 order (Table S1 and Fig. 2). To our knowledge, there have been no reports illustrating the structures of PGCs in SG8-23 or UBA6960. In this study, we assembled from the metagenomes of Qinghai Lake sediments three PGC-containing genomes of the UBA6960 family (N1_bin156, N4_bin48, and N5_bin42) and a PGC-containing genome of *Gemmatimonadaceae* (N4_bin22).

**FIG 4:**
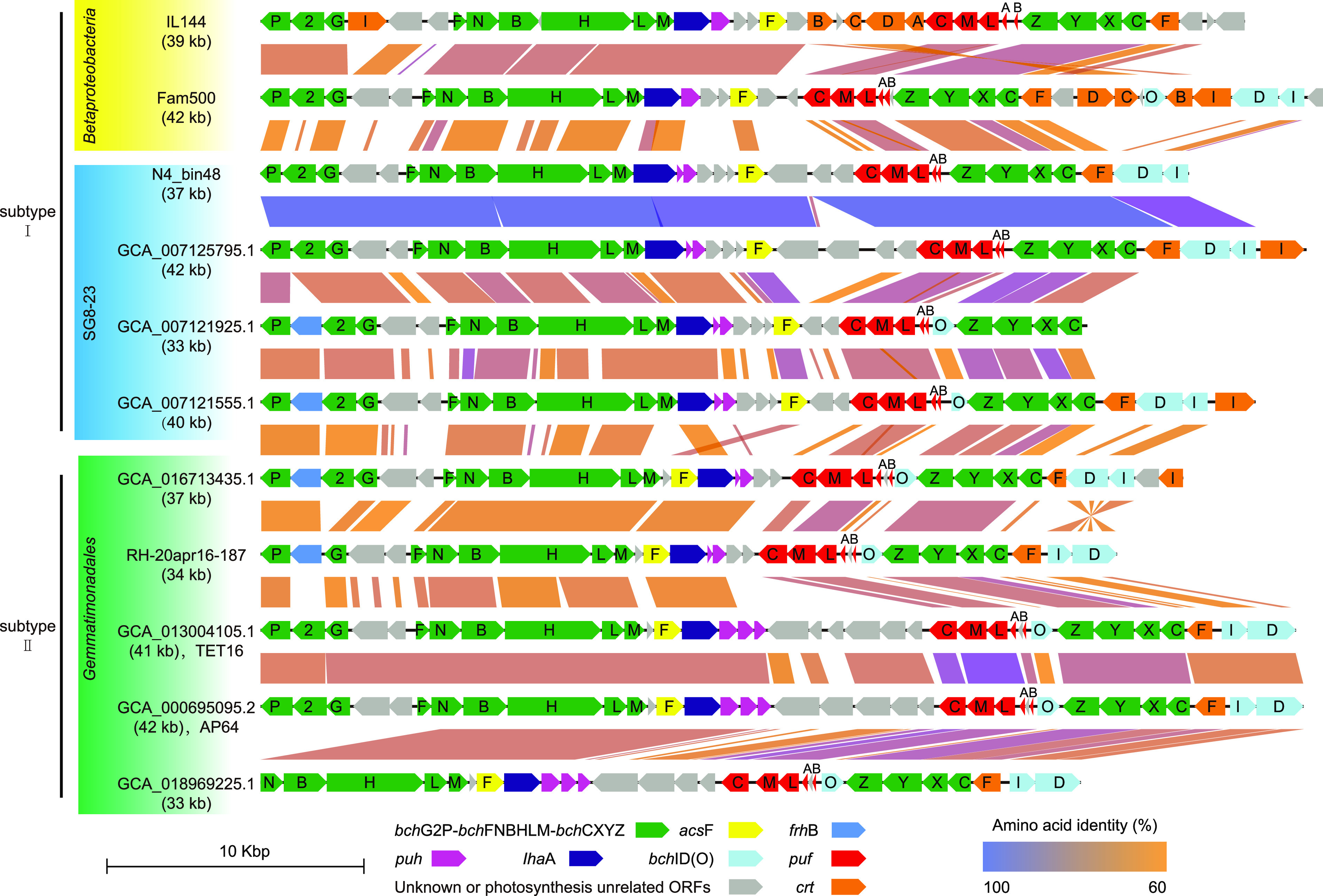
The PGC organization in *Gemmatimonadota*. *bch*, bacteriochlorophyll biosynthesis genes; *acs*F, encoding magnesium-protoporphyrin IX monomethyl ester (oxidative) cyclase; *puh*, genes encoding reaction center assembly proteins; *Iha*A, encoding reaction center assembly proteins; *puf*, genes encoding reaction center proteins; *crt*, carotenoid biosynthesis genes; *frh*B, encoding coenzyme F420-reducing hydrogenase beta subunit. PGC (photosynthesis gene cluster) in MAG of GCA_007125795.1 (CSSed162cmB_505) was actually joined from three contigs; however, it was almost 100% similar with the PGC in only one contig from the MAG of GCA_007120305.1 (CSSed162cmA_30R1). The ANI value of these two MAGs was almost 100%, indicating they belong to the same species. Given a higher completeness and lower contamination of the MAG of GCA_007125795.1, we removed the redundant MAG of GCA_007120305.1.

PGC genes in several genomes are located in different contigs or scaffolds, presumably as a result of the fragmented nature of metagenome assemblies. We chose nine PGCs, which are located in the same contig or scaffold, for further analysis. Although the PGCs of N5_bin42 are on a single contig and the PGCs of N1_bin156 and N4_bin48 are obtained by joining two contigs, the former is 100% identical to the latter two when the homologous sequences are compared (Fig. S3). Therefore, the PGCs from N4_bin48, the longest among the three genomes, was used in gene arrangement comparison with those from genomes in public databases (Fig. 4). PGCs from the genomes of two betaproteobacteria, Methyloversatilis universalis Fam500 and Rubrivivax gelatinosus IL-144, were used as references (13, 21).

All of the 11 selected PGCs contain three conserved gene clusters, i.e., *bch*FNBHLM, *puf*BALMC, and *crt*F-*bch*CXYZ, and belong to type II PGCs since *bchF*NBHLM and *crt*F-*bch*CXYZ are transcribed in opposite orientations (Fig. 4) (42). Based on the relative locations of *acs*F (encoding magnesium-protoporphyrin IX monomethyl ester [oxidative] cyclase) and *Iha*A (encoding reaction center assembly proteins), they can be further divided into two subgroups, i.e., subtype I, in which *acs*F is located downstream of *Iha*A and the two genes are separated by *puh* (encoding reaction center assembly proteins) as well as unannotated genes, and subtype II, in which *acs*F is upstream of and adjacent to *Iha*A. All of the 10 PGCs from the SG8-23 genomes belong to subtype I, and all of the remaining 32 PGCs, except for one (S09.Bin022, GCA_011390645.1), are of subtype II (Table S2).

Among the *bch* genes encoding the biosynthesis of bacteriochlorophyll a (BChl a), *bch*IDO (genes coding for magnesium chelatase ATPase subunits I and D and putative accessory protein O) are arranged in distinctly different manners in the PGCs (Fig. 4). The *bch*ID genes, which are upstream of *crt*F in PGCs from SG8-23, are normally transcribed in the same direction as the carotenoid synthesis gene (*crt*F). In comparison, the direction of transcription of *bch*ID is reversed in PGCs from some *Gemmatimonadales*, such as AP64 and TET16. *bch*O from both SG8-23 and *Gemmatimonadales*, generally located between *bch*CXYZ and *puf*ABLMC (encoding reaction center proteins), is transcribed in a direction opposite to that of the conserved *bch*CXYZ operon. Notably, *bch*O appears to be absent from some PGCs from both SG8-23 and *Gemmatimonadales* genomes (Fig. 4). Since *frh*B, which encodes the coenzyme F420-reducing hydrogenase beta subunit (EC:1.12.98.1), is found in both SG8-23 and *Gemmatimonadales* PGCs (e.g., GCA_007121925.1, GCA_007121555.1, GCA_016713435.1, and RH-20apr16-187), it could potentially replace *bch*2 (a 4.5-kDa chain of bacteriochlorophyll synthase) and form a unique *bch*P-*frh*B-*bch*G operon for BChla biosynthesis (Fig. 4) (4). Furthermore, a region highly variable with respect to the number of hypothetical genes or genes unrelated to photosynthesis as well as the direction of transcription of the genes exists between the *puh* and *puf*BALMC operons (Fig. 4). These observations suggest that PGCs have undergone a complex process of recombination following their divergence from a common ancestor, and two evolutionary pathways for photosynthesis have occurred within *Gemmatimonadota*.

### Evolution of photosynthesis genes in *Gemmatimonadota*.

Phototrophs include both retinalophototrophs and chlorophototrophs, which employ retinalrhodopsin and chlorophyll, respectively, to harvest light energy (43–45). Chlorophototrophs are currently found in seven bacterial phyla: *Cyanobacteria*, *Proteobacteria* (purple anoxygenic phototrophs), *Chlorobi* (green sulfur bacteria), *Chloroflexi* (green nonsulfur bacteria), *Firmicutes* (heliobacteria), *Acidobacteria*, and *Gemmatimonadota* (45). Aside from *Cyanobacteria*, chlorophototrophs from the remaining six phyla are anoxygenic phototrophic bacteria (APB) that harvest light using various forms of bacteriochlorophyll (*bch*) without oxygen production through water oxidation. Most APBs are found in restricted habitats. For example, *Chlorobi* species exist mainly in anoxic aquatic habitats (46), *Chloroflexi* species in low-oxygen hot springs (47–49), and the only Gram-positive APB (heliobacteria) in soil (50). In contrast, the APB of *Gemmatimonadota* appear to have a more cosmopolitan distribution (4, 16, 51).

**FIG 5:**
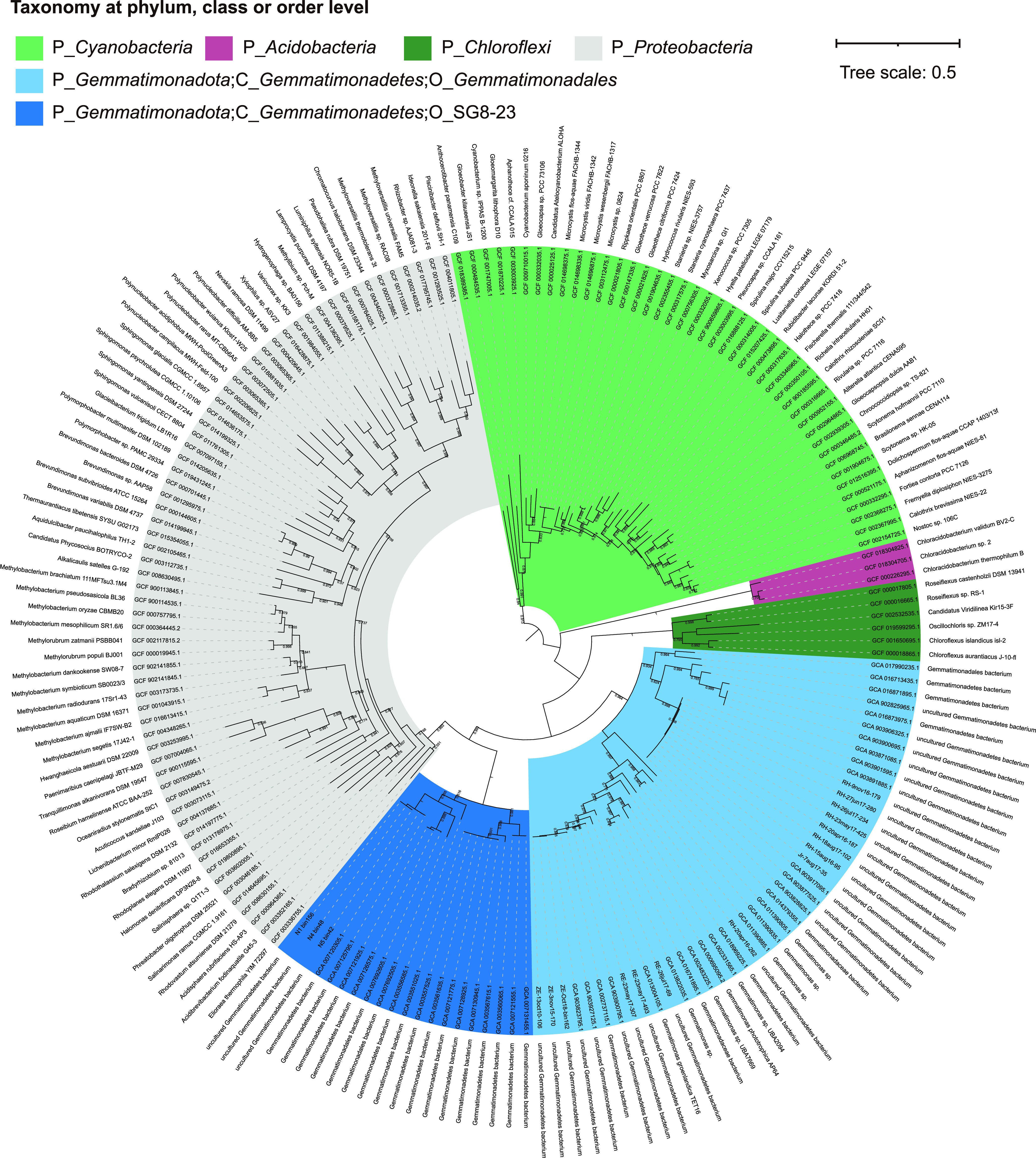
Phylogenetic analysis of *Gemmatimonadota acs*F-like proteins in relation to other phototrophic bacterial phyla.

To explore the evolutionary relationships of phototrophic *Gemmatimonadota*, proteins encoded by *acs*F, *bch*H, and *puf*LM in 73 genomes (Table S2) and selected homologous sequences from other phyla were used to construct phylogenetic trees (Fig. 5 and Fig. S4). AcsF, the aerobic magnesium-protoporphyrin IX monomethyl ester (oxidative) cyclase, is an essential protein for BChla biosynthesis in all phototrophs and thus widely used in the phylogenetic analysis of phototrophs (13, 15–17, 52). BchH is the largest protein (~1,271 amino acids) encoded in the relatively conserved region (*bch*FNBHLM), whereas PufLM are the subunits of the photosynthetic reaction center. *Gemmatimonadales* and SG8-23 form two separate clusters, and they are clearly separated from the main chlorophototrophic groups, i.e., *Cyanobacteria*, *Chloroflexi*, *Acidobacteria*, *Firmicutes*, *Chlorobi*, and *Proteobacteria*, as revealed by the AcsF and BchH trees (Fig. 5 and Fig. S4). However, not all *Gemmatimonadales* cluster together in the PufL or PufM tree (Fig. S4), presumably due to frequent genetic exchanges as observed in the PGC structures. In all of the four phylogenetic trees (Fig. 5 and Fig. S4), *Gemmatimonadota* and *Proteobacteria* cluster in the same clade, demonstrating a common evolutionary origin of their photosystem. This supports the hypothesis that *Gemmatimonadota* acquired PGC via horizontal gene transfer (HGT) from *Proteobacteria* (13). However, it is still unclear what kind of HGT, e.g., transformation, transduction, or conjugation, is responsible for acquiring foreign PGCs across phyla. It has been reported that extrachromosomal replicons may play an important role in the transfer of PGCs in bacteria (53). Further, SG8-23 is more closely related to *Proteobacteria* than to *Gemmatimonadales* in the AcsF tree (Fig. 5), whereas *Gemmatimonadales*, and not SG8-23, is clustered with *Proteobacteria* in the same clade in the BchH tree (Fig. S4), suggesting PGC genes may have evolved at different rates in the two orders.

Taken together, our results suggest that the photosystem was acquired by the ancestor of *Gemmatimonadota* before it diverged into *Gemmatimonadales*, SG8-23, and other phototrophic orders. This suggestion is supported by high topological congruence between the organismal and PGC genes-based phylogenies of *Gemmatimonadota*. The acquired PGC genes are passed on in at least two independent routes, leading to subtypes I and II PGCs in *Gemmatimonadota*. Nonphototrophic *Gemmatimonadota* may have lost their PGC genes during the course of evolution.

### Conclusions.

*Gemmatimonadota* are globally distributed in various habitats, and both chemotrophic and phototrophic growth types are widely adopted by its members. Our genomic analysis suggests that *Gemmatimonadota* are capable of degrading various complex organic substrates and pursuing a heterotrophic pathway (e.g., glycolysis and TCA cycle) for their growth. Diverse strategies for energy conservation, including oxidative phosphorylation, substrate phosphorylation, and photosynthetic phosphorylation, are employed by *Gemmatimonadota*. And the processes of sufficient energy being stored in glucose through gluconeogenesis, followed by the synthesis of more complex compounds (e.g., glycogen and starch), are also prevalent in this phylum. Most members of the phylum are able to perform aerobic respiration. After diverging from the ancestor of *Gemmatimonadota*, the SG8-23 branch has become more suited to growth in saline environments, in agreement with the fact that most of the assembled genomes in this branch were originally from marine and saline soda habitats, than *Gemmatimonadales*. Moreover, the photosystem in *Gemmatimonadota* has evolved in two independent routes.

## MATERIALS AND METHODS

### Sample collection, DNA extraction, and high-throughput sequencing.

Three sediment samples, denoted N1, N4, and N5, were collected from Qinghai Lake, a perennial salt lake located in a structural intermontane depression at the northeastern corner of the Qinghai–Tibetan Plateau (54, 55), China, in June 2018. Each sample was placed in a sterile plastic bag using a sterile scoop and immediately stored at 4°C. The GPS coordinates of the sampling sites are listed in Data Set S1, Sheet 1. A fraction (10 g by wet weight) of each sample was subjected to DNA extraction as described previously (23, 56). DNA was purified by using a PowerClean Pro DNA Clean-Up Kit (MO BIO Laboratories), sheared to 400~500 bp in size with the Covaris M220 Focused-Ultrasonicator and quantified by the Agilent 2100 bioanalyzer (Agilent Technologies Inc., USA). The sequencing libraries were constructed using the KAPA Hyper Prep Kit (Kapa Biosystems). Paired-end (PE) sequencing (2 × 250 bp) was conducted on an Illumina Hiseq-2500 platform at Beijing Institute of Genomics, Chinese Academy of Sciences (CAS), Beijing, China. Deep-sea sediments (TVG05 and TVG06), from the Southwest Indian Ocean during the DY125-39 cruise with R/V Dayang No.1, have been previously described by Zheng et al. (23).

### Metagenome assembly and genome binning.

Raw reads from each sample were adapter trimmed, and low-quality reads were removed using fastp (v0.19.4) with default parameters (57). The clean PE reads from each sample were first merged using BBMerge (58) and separately assembled by SPAdes (v3.13.0, --meta --only-assembler) with a series of k-mers (i.e., 21, 31, 55, 77, 99, 121) (59). Contigs over 5 kb in length from each sample were selected for binning analysis using MetaBAT2 (v2.12.1) (60, 61) and MaxBin2 (v2.2.5) (62, 63) modules in MetaWRAP pipeline (v1.0.3) with default parameters (64). The resulting MAGs were refined by the bin_refinement module in the Binning_refiner (v1.2) (65) to filter out duplicate contigs and merge similar MAGs. The completeness, potential contamination, and strain heterogeneity of MAGs were evaluated via CheckM (v1.0.9) with lineage-specific marker genes (66). Taxonomic assignments of the MAGs were verified with GTDB-Tk (v0.3.3) (67, 68). Only medium- to high-quality (completeness ≥50% and contamination ≤5%) MAGs belonging to phylum *Gemmatimonadota* were retained for further analysis. The genome size of each MAG was estimated as described by Chen et al. (69). 16S rRNA gene tags were identified from merged reads and clustered against the SILVA database as described previously (23). Briefly, 16S rRNA gene tags were identified using the script “rna_hmm.py” (70, 71). And the resulting sequences were assigned (97% identity) to SILVA reference OTUs (release 138: SSU Ref NR 99) using UCLUST (71–73).

### Phylogenetic analysis.

Published genomes and MAGs of *Gemmatimonadota* (1, 12–15, 74), verified by GTDB-Tk (68), were retrieved from the NCBI Assembly database (https://ftp.ncbi.nlm.nih.gov/genomes/all/GCA/, July 2021) and Genome Taxonomy Database (GTDB, https://gtdb.ecogenomic.org/). Forty-five *Gemmatimonadota* MAGs of the freshwater origin, as reported by Mujakic et al. (4), were downloaded from Figshare (https://figshare.com/). To reduce redundancy, all above genomes were aggregated and dereplicated at 99% average nucleotide identity (ANI) using dRep v2.3.2 (parameters: -comp 50 -con 5 -sa 0.99) (24), resulting in a total of 326 strain-level genomes. These genomes were also dereplicated at 95% ANI to calculate the number of species represented. Values of orthologous average nucleotide identity (OrthoANI) and average amino acid identity (AAI) were calculated by using OrthoANIu (75) and EzAAI (76), respectively. Phylogenetic analysis of the above nonredundant genomes based on 120 bacterial marker proteins were conducted using the “identify” and “align” steps in GTDB-Tk (68). Maximum likelihood (ML) phylogeny was inferred with FastTree v.2.1.10 in the WAG+GAMMA model (77). The phylogenetic tree was visualized on the iTOL (78).

### Functional annotation and metabolic reconstruction.

Open reading frames (ORFs) in the above nonredundant genomes were predicted using Prokka (79) with default parameters. All ORFs were annotated using the KEGG database with GhostKOALA (80), eggNOG 5.0 using eggNOG-mapper 2 (80, 81), and Pfam 31.0 (82) using hmmsearch with a bit score of 30 (83). The MAGs and downloaded genomes were also submitted to the METABOLIC v2.0 annotation pipeline for functional annotation (84). Potential metabolic pathways were reconstructed based on these annotations. A hypergeometric test through the R package phytools (85) was applied to infer the metabolic differences among various *Gemmatimonadota* groups. ORFs encoding bacteriochlorophyll biosynthesis (acsF and bchH) and subunits of the photosynthetic reaction center complex (pufL and pufM) were aligned individually against proteins downloaded from the NCBI RefSeq genome database (https://ftp.ncbi.nlm.nih.gov/genomes/all/GCF/, July, 2021) using Diamond (v0.9.10.111) with default parameter (86). Target proteins with the highest BLASTp scores at class level were selected for phylogeny analysis.

### Pangenome and core genome analysis of *Gemmatimonadota*.

A *Gemmatimonadota* pangenome is defined as the total number of protein-coding gene families found in all of the *Gemmatimonadota* genomes and MAGs. Since unknown portions of genes are missing from those incomplete genomes, a core genome of *Gemmatimonadota* is defined as all of the protein-coding gene families found in 95% of the genomes and high-quality MAGs (completeness ≥90% and contamination ≤5%). Gene families of the above ORFs were clustered using the get_homologues package (v3.4.3) (87) based on “diamond blastp” and “OMCL” algorithms with default parameters. Gene accumulation curves, which describe the sizes of the pan-genome and the core genome, were plotted using R package ggplot2 after adding new genome data during 100 random duplications (88). A gene content matrix consisting of the number of pan-genome orthologs in each genome was used to determine the relationship between each pair of genomes by deriving a correlation coefficient value (Pearson’s coefficient) using the “cor” function in the R program, and was further visualized using the “pheatmap” function for genomic similarity comparison (89).

### Data availability.

Metagenomic raw reads are accessible in NODE (https://www.biosino.org/node/) with accession number OEP001438, and in NCBI (https://www.ncbi.nlm.nih.gov/bioproject) under the BioProject numbers PRJNA776043 and PRJNA573810. The genome sequences of 17 MAGs retrieved in this study have been deposited in eLMSG (an eLibrary of Microbial Systematics and Genomics, https://www.biosino.org/elmsg/index) under accession numbers LMSG_G000003454.1–LMSG_G000003470.1.

10.1128/msystems.00228-22.1 DATA SET S1 Sheet 1. Statistics of sequencing and assembly results. Sheet 2. Information on samples and MAGs. Download Data Set S1, XLSX file, 0.01 MB. Copyright © 2022 Zheng et al. 2022 Zheng et al. https://creativecommons.org/licenses/by/4.0/ This content is distributed under the terms of the Creative Commons Attribution 4.0 International license.

10.1128/msystems.00228-22.2 DATA SET S2 Sheet 1. Relative abundance (%) of microorganisms in different Qinghai Lake sediments at the domain and phylum levels based on 16S _mi_tags. Sheet 2. Relative abundance (%) of *Gemmatimonadota* in EMP samples based on 16S rRNA reads. Download Data Set S2, XLSX file, 1.5 MB. Copyright © 2022 Zheng et al. 2022 Zheng et al. https://creativecommons.org/licenses/by/4.0/ This content is distributed under the terms of the Creative Commons Attribution 4.0 International license.

10.1128/msystems.00228-22.3 DATA SET S3 Sheet 1. Hypergeometric test based on KEGG functional categories for the genomes of *Gemmatimonadales* and SG8-23 (completeness ≥90% and contamination ≤5%). Sheet 2. Hypergeometric test based on KEGG orthology (KO) annotations for the genomes (completeness ≥90% and contamination ≤5%) of SG8-23 and *Gemmatimonadales* orders. Sheet 3. Hypergeometric test based on KEGG orthology (KO) annotations for the genomes (completeness ≥90% and contamination ≤5%) of UBA6960 and *Gemmatimonadaceae*, GWC2-71-9 families. Sheet 4. Hypergeometric test based on KEGG orthology (KO) annotations for the genomes (completeness ≥90% and contamination ≤5%) of GWC2-71-9 and UBA6960, *Gemmatimonadaceae* families. Sheet 5. Hypergeometric test based on KEGG orthology (KO) annotations for the genomes (completeness ≥90% and contamination ≤5%) of *Gemmatimonadaceae* and UBA6960, GWC2-71-9 families. Download Data Set S3, XLSX file, 0.03 MB. Copyright © 2022 Zheng et al. 2022 Zheng et al. https://creativecommons.org/licenses/by/4.0/ This content is distributed under the terms of the Creative Commons Attribution 4.0 International license.

10.1128/msystems.00228-22.4 DATA SET S4 Sheet 1. Carbohydrate-active enzymes (CAZymes) annotated in 326 nonredundant *Gemmatimonadota* genomes. Sheet2. KEGG pathway and related metabolism of the 326 nonredundant *Gemmatimonadota* genomes. Sheet 3. Genomes potentially encoding both aerobic respiration and anaerobic respiration using NO, N_2_O, NO_3_^–^ or NO_2_^–^ as terminal electron acceptors. Download Data Set S4, XLSX file, 0.4 MB. Copyright © 2022 Zheng et al. 2022 Zheng et al. https://creativecommons.org/licenses/by/4.0/ This content is distributed under the terms of the Creative Commons Attribution 4.0 International license.

10.1128/msystems.00228-22.5 TABLE S1 Detailed information of all the 521 *Gemmatimonadota* genomes or MAGs. Download Table S1, XLSX file, 0.1 MB. Copyright © 2022 Zheng et al. 2022 Zheng et al. https://creativecommons.org/licenses/by/4.0/ This content is distributed under the terms of the Creative Commons Attribution 4.0 International license.

10.1128/msystems.00228-22.6 TABLE S2 Gene order of PGC in 73 potential phototrophic *Gemmatimonadota* genomes. Download Table S2, XLSX file, 0.02 MB. Copyright © 2022 Zheng et al. 2022 Zheng et al. https://creativecommons.org/licenses/by/4.0/ This content is distributed under the terms of the Creative Commons Attribution 4.0 International license.

10.1128/msystems.00228-22.7 FIG S1 A heatmap of correlation coefficient values (Pearson’s coefficient) showing similarities between each pair of genomes (≥90% completeness, ≤5% contamination) in SG8-23 and *Gemmatimonadales*. A gene content matrix consisting of the number of pan-genome orthologs in each genome was used to determine the relationship between each pair of genomes by deriving a correlation coefficient value (Pearson’s coefficient). Highly correlated genomes are clustered together. Both *x* and *y* axes represent the IDs of the genomes. Download FIG S1, PDF file, 1.0 MB. Copyright © 2022 Zheng et al. 2022 Zheng et al. https://creativecommons.org/licenses/by/4.0/ This content is distributed under the terms of the Creative Commons Attribution 4.0 International license.

10.1128/msystems.00228-22.8 FIG S2 Accumulation curves describing the numbers of core-genome and pan-genome gene families for the 143 *Gemmatimonadota* genomes (A, ≥90% completeness, ≤5% contamination), and 326 *Gemmatimonadota* genomes (B, ≥50% completeness, ≤5% contamination). Download FIG S2, PDF file, 0.3 MB. Copyright © 2022 Zheng et al. 2022 Zheng et al. https://creativecommons.org/licenses/by/4.0/ This content is distributed under the terms of the Creative Commons Attribution 4.0 International license.

10.1128/msystems.00228-22.9 FIG S3 The PGC organization in *Gemmatimonadota* MAGs assembled from the three metagenomes of Qinghai Lake sediments. *bch*, bacteriochlorophyll biosynthesis genes; *acs*F, encoding magnesium-protoporphyrin IX monomethyl ester (oxidative) cyclase; *puh*, genes encoding reaction center assembly proteins; *Iha*A, encoding reaction center assembly proteins; *puf*, genes encoding reaction center proteins; *crt*, carotenoid biosynthesis genes; *frh*B, encoding coenzyme F420-reducing hydrogenase beta subunit. Download FIG S3, PDF file, 0.4 MB. Copyright © 2022 Zheng et al. 2022 Zheng et al. https://creativecommons.org/licenses/by/4.0/ This content is distributed under the terms of the Creative Commons Attribution 4.0 International license.

10.1128/msystems.00228-22.10 FIG S4 Phylogenetic analysis of *Gemmatimonadota* bchH-like proteins (A), photosynthetic reaction center subunit pufL (B), and pufM (C) among *Gemmatimonadota* and other phototrophic bacterial phyla. Download FIG S4, PDF file, 2.0 MB. Copyright © 2022 Zheng et al. 2022 Zheng et al. https://creativecommons.org/licenses/by/4.0/ This content is distributed under the terms of the Creative Commons Attribution 4.0 International license.
