# Supplementary figures and images for: (Meta)Genomic Analysis Reveals Diverse Energy Conservation Strategies Employed by Globally Distributed Gemmatimonadota
Source: mSystems. 2022 Aug 1;7(4):e00228-22. doi: 10.1128/msystems.00228-22 (PMC9426454; doi:10.1128/msystems.00228-22)

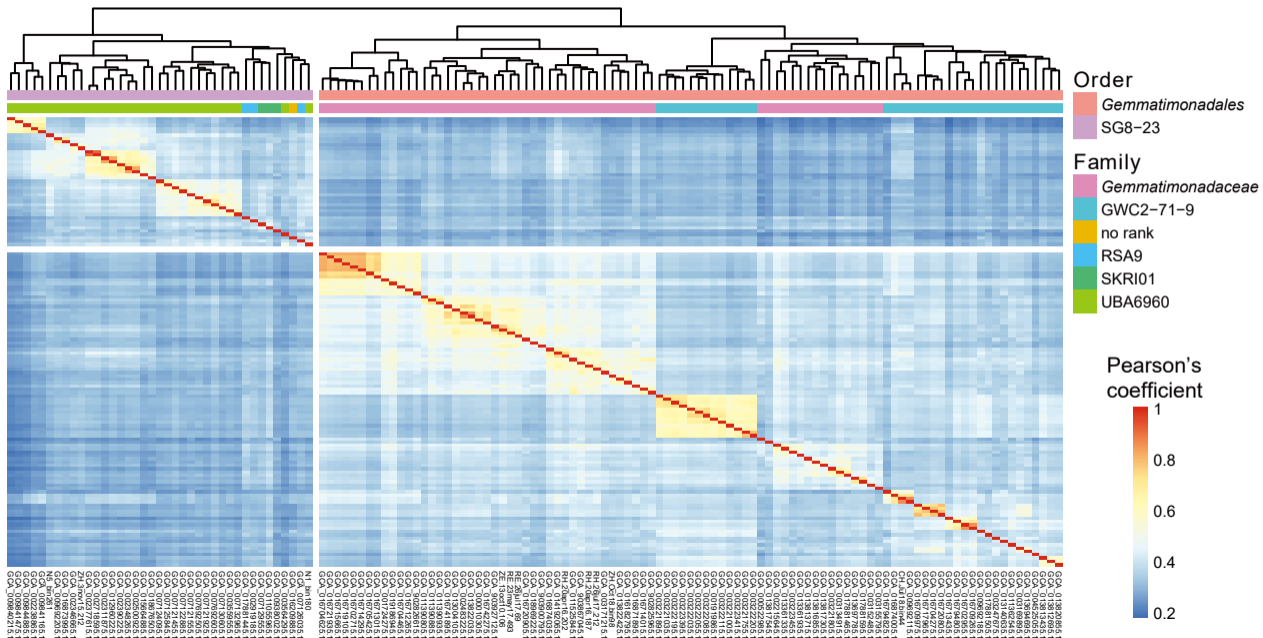

Supplement: FIG S1 [file msystems.00228-22-s0007.pdf]

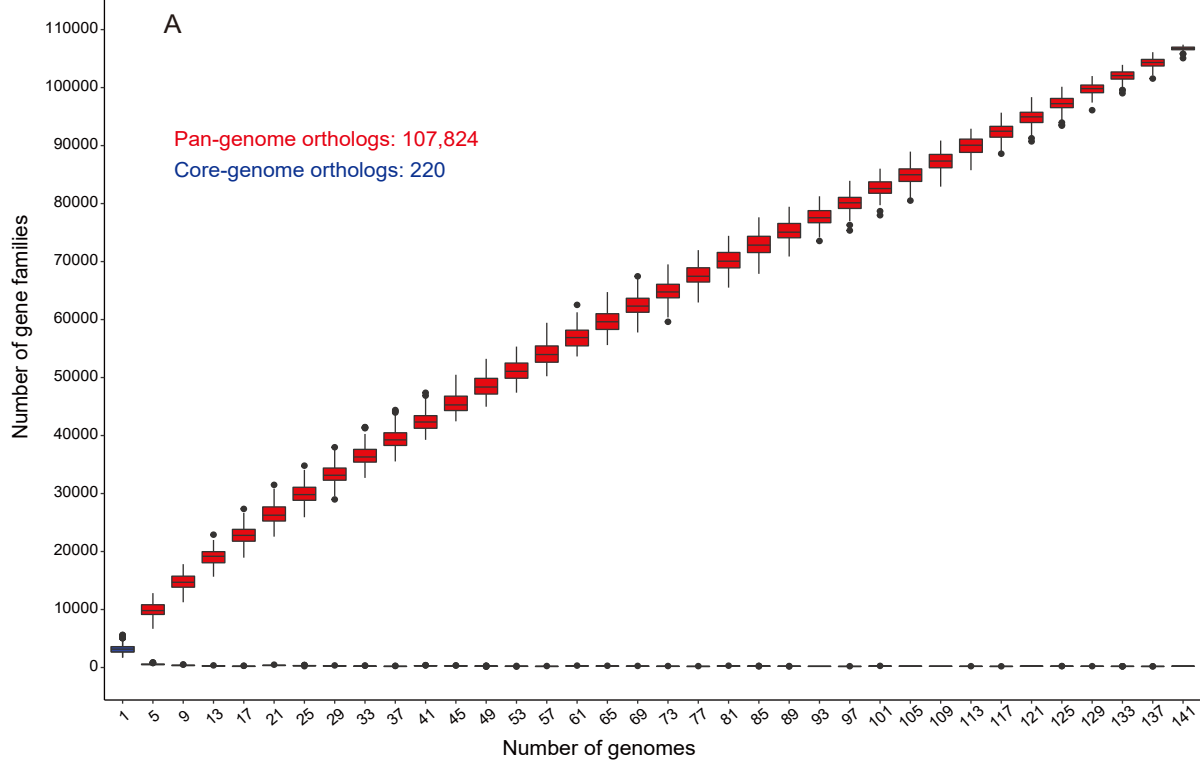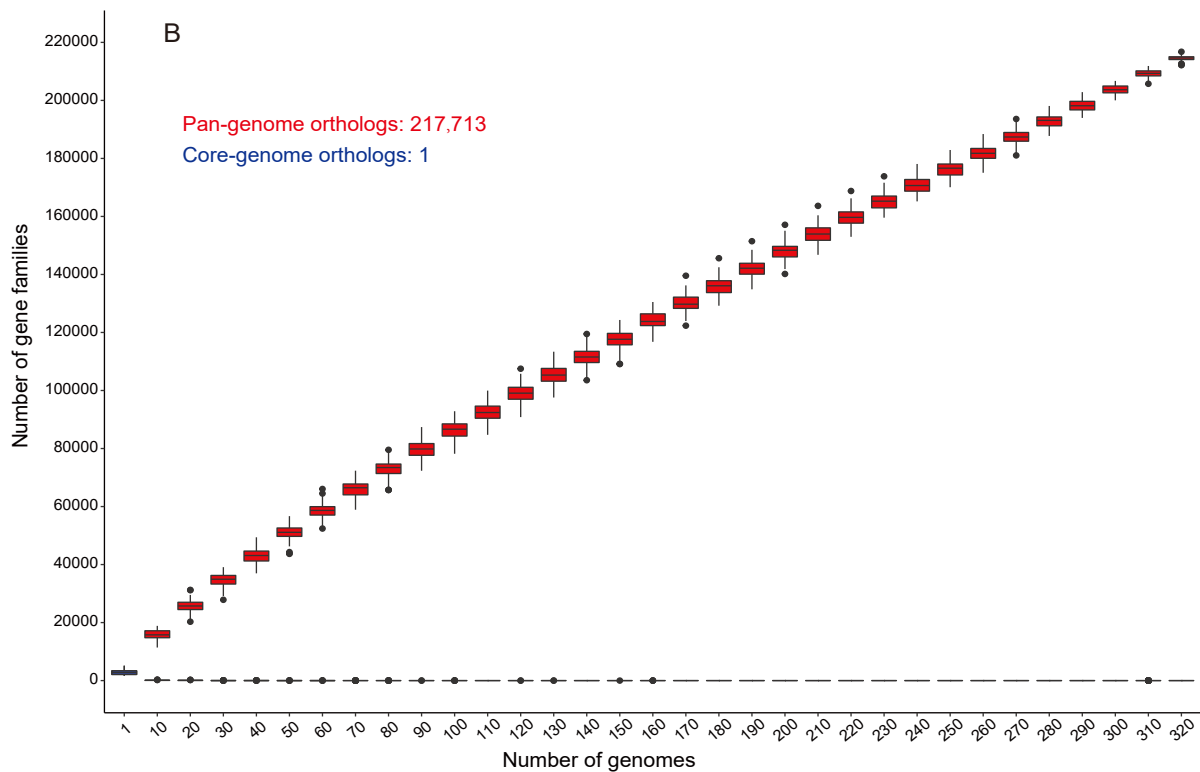

Supplement: FIG S2 [file msystems.00228-22-s0008.pdf]

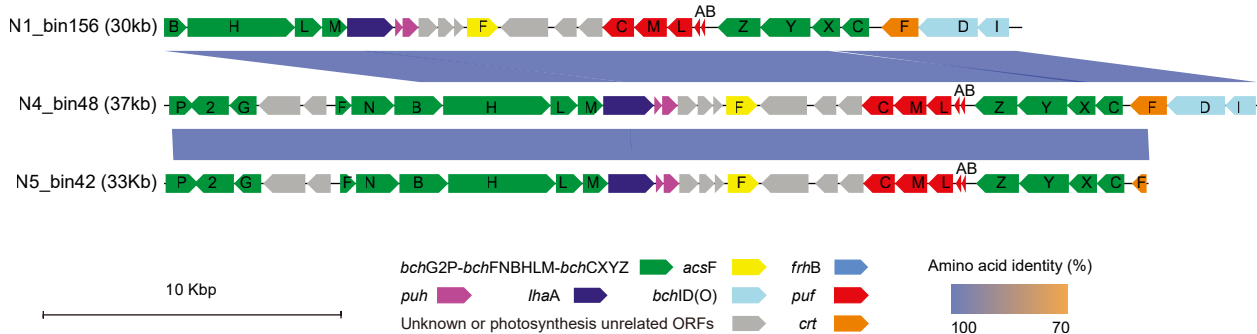

Supplement: FIG S3 [file msystems.00228-22-s0009.pdf]
