# Supplementary material for: (Meta)Genomic Analysis Reveals Diverse Energy Conservation Strategies Employed by Globally Distributed Gemmatimonadota
Source: mSystems. 2022 Aug 1;7(4):e00228-22. doi: 10.1128/msystems.00228-22 (PMC9426454; doi:10.1128/msystems.00228-22)
Supplement: FIG S4 [file msystems.00228-22-s0010.pdf]

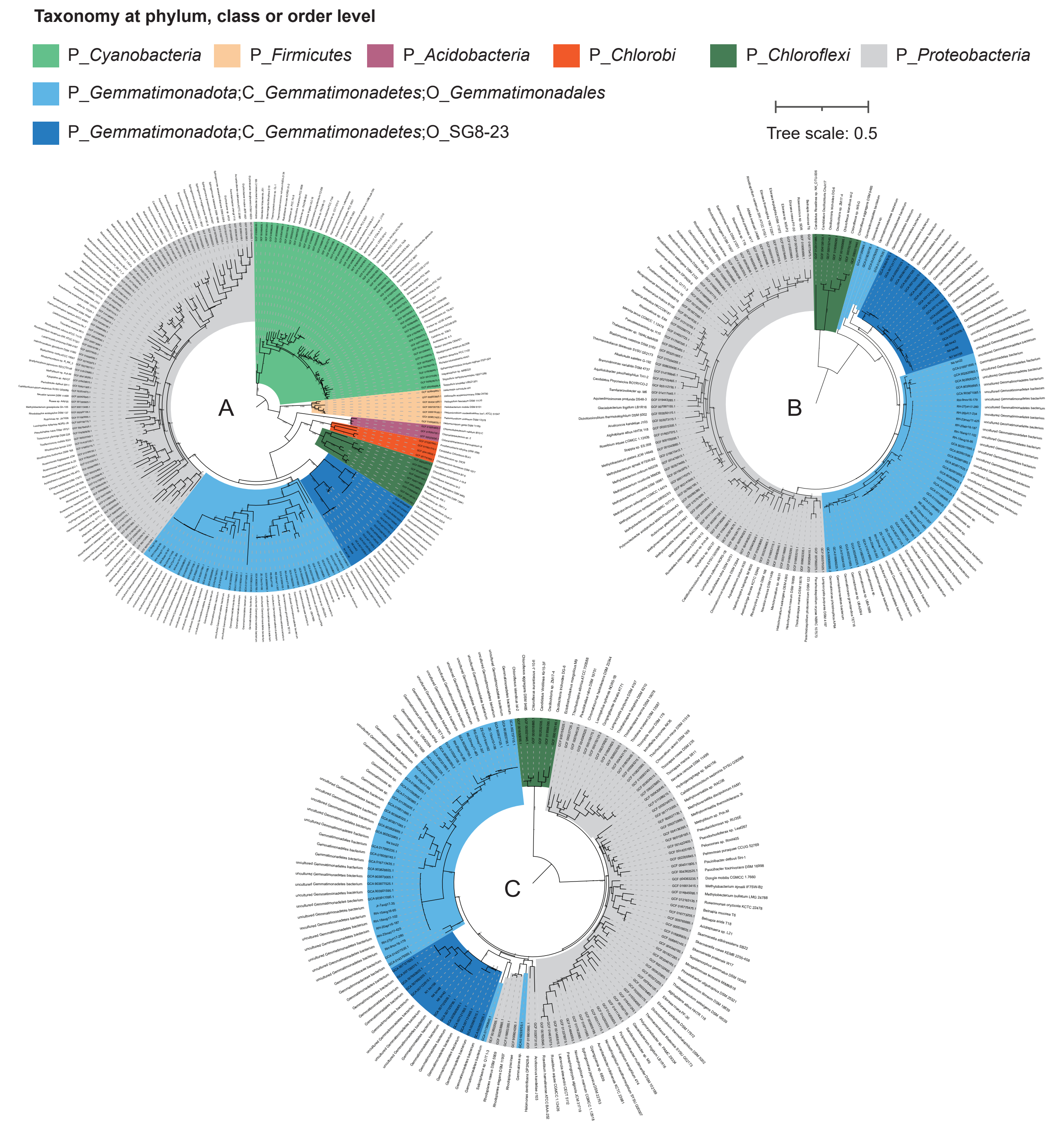
Taxonomy at phylum, class or order level
P_Cyanobacteria
P_Firmicutes
P_Acidobacteria
P_Chlorobi
P_Chloroflexi
P_Proteobacteria
P_Gemmatimonadota;C_Gemmatimonadetes;O_Gemmatimonadales
P_Gemmatimonadota;C_Gemmatimonadetes;O_SG8-23
Tree scale: 0.5
A
B
C
